# Indiana State Board of Health

**Published:** 1886-01

**Authors:** 


INDIANA STATE BOARD OF HEALTH.

INDIANA MEDICAL PRACTICE ACT.

The quarterly report of the Illinois State Board of Health says:

"An act regulating the practice of medicine, surgery and obstetrics was passed by the last Legislature of Indiana, the same going into effect on the 23d of July.

"While the law is better than none, it is yet very defective in that it leaves the county clerks the judges of the reputable character of Medical Colleges, thus placing a most important trust in the hands of officials who, however honest they may be, cannot have the special acquirements necessary to judge correctly regarding a Medical School's standing; and it is to be feared that this provision of the law will stimulate the graduation of incompetent individuals."

